# Grid-Based Clustering Using Boundary Detection

**DOI:** 10.3390/e24111606

**Published:** 2022-11-04

**Authors:** Mingjing Du, Fuyu Wu

**Affiliations:** School of Computer Science and Technology, Jiangsu Normal University, Xuzhou 221116, China

**Keywords:** grid-based clustering, density-based clustering, boundary detection

## Abstract

Clustering can be divided into five categories: partitioning, hierarchical, model-based, density-based, and grid-based algorithms. Among them, grid-based clustering is highly efficient in handling spatial data. However, the traditional grid-based clustering algorithms still face many problems: (1) Parameter tuning: density thresholds are difficult to adjust; (2) Data challenge: clusters with overlapping regions and varying densities are not well handled. We propose a new grid-based clustering algorithm named GCBD that can solve the above problems. Firstly, the density estimation of nodes is defined using the standard grid structure. Secondly, GCBD uses an iterative boundary detection strategy to distinguish core nodes from boundary nodes. Finally, two clustering strategies are combined to group core nodes and assign boundary nodes. Experiments on 18 datasets demonstrate that the proposed algorithm outperforms 6 grid-based competitors.

## 1. Introduction

Clustering is the process of grouping similar objects together [1]. As a fundamental data mining task, it can be used either independently or as a preprocessing step before other data mining tasks. Clustering plays an important role in many scientific fields [2], including earth sciences [3,4], biology [5,6,7], and economics [8,9].

Generally, clustering can be divided into five categories: partitioning [10,11], hierarchical [12,13], model-based [14,15], density-based [16,17,18], and grid-based algorithms. Partitioned clustering is designed to discover clusters in the data by optimizing a given objective function. Hierarchical clustering deals with the clustering problem by constructing a tree diagram. Model-based clustering uses a probabilistic methodology to optimize the match between some mathematical models and the data. Density-based and grid-based solutions are two closely related categories that attempt to explore the data space at a high level of granularity.

In recent decades, many grid-based clustering algorithms have been developed. In these algorithms, the data space is partitioned into a finite number of cells to form a grid structure. Clusters correspond to regions that are the connected cells with more density. As most grid-based clustering algorithms rely on calculations of cell density, these algorithms may be considered density-based. Even some grid-based clustering algorithms are developed by improving density-based clustering. Among density-based clustering algorithms, DBSCAN [19] and DPC [20] algorithms are the most widely used and have the most variants.

The computation cost of grid-based clustering is determined by the number of grid cells, independent of dataset size. Generally, grid-based clustering is more efficient than other clustering algorithms for large-scale spatial data since the number of cells is significantly smaller than the number of data points. Although grid-based clustering algorithms greatly improve computational efficiency, they still face some of the following problems.

- They are sensitive to the parameter of density threshold, which may be difficult to obtain.
- They may not be sufficient to achieve desired clustering results for data with varying densities.
- They are not able to handle boundary regions between some adjacent clusters well.

We aim at alleviating the above-mentioned issues and propose a grid-based clustering using boundary detection, named GCBD. Firstly, the density estimation of nodes is defined using the standard grid structure. Secondly, GCBD uses an iterative boundary detection strategy to distinguish core nodes from boundary nodes. Finally, DBSCAN and DPC clustering strategies are combined to group core nodes and assign boundary nodes.

The rest of this paper is organized as follows. We survey the related work in Section 2. Our proposed GCBD algorithm is presented in Section 3. Section 4 presents the experimental results. Conclusions and suggestions for future work are given in Section 5.

## 2. Related Work

### 2.1. Grid-Based Clustering

In this subsection, we discuss some classical grid-based clustering algorithms. To our knowledge, Schikuta [21] introduces the first grid-based hierarchical clustering algorithm called GRIDCLUS. In GRIDCLUS, data points are designated to blocks in the grid structure such that their topological distributions are maintained. GRIDCLUS calculates the density for each block. The blocks are clustered iteratively in order of descending density to form a nested sequence of nonempty, disjoint clusters. Schikuta and Erhart [22] further propose the BANG algorithm to improve the inefficiency of GRIDCLUS in terms of grid structure size and neighbor search. Wang et al. [23] propose a statistical information grid-based clustering method (STING) to cluster spatial data. In contrast to GRIDCLUS and BANG, STING divides the spatial area into rectangular cells and uses a hierarchical grid structure for storing the cells. Using the hierarchical grid structure may generate rougher cluster boundaries, which reduces clustering quality. As a solution to the problem, Sheikholeslami et al. [24] propose WaveCluster, a grid-based and density-based clustering approach utilizing wavelet transforms. In this algorithm, wavelet transforms are applied to the spatial data feature space to detect arbitrary shape clusters at different scales.

A significant issue with grid-based algorithms is their scalability in higher dimensional data since the time complexity grows as the number of grids increases. To address the challenge, Agrawal et al. [25] invent the algorithm CLIQUE (clustering in quest). CLIQUE seeks dense rectangular cells in all subspaces with high density by applying a bottom-up scheme. Clusters are generated as the connected areas of dense cells. OptiGrid (optimal grid clustering) [26] significantly modifies CLIQUE. OptiGrid constructs the best cutting hyperplanes through a set of projections to obtain optimal grid-partitioning. The above algorithm is very sensitive to the density threshold and it is difficult to adjust this parameter.

In the grid-based clustering, variants of the DBSCAN algorithm [19] are the most closely related to our algorithm. Wu et al. [27] propose a density- and grid-based (DGB) clustering algorithm inspired by grid partitioning and DBSCAN. The DGB algorithm only needs to calculate distances between grid nodes instead of distances between data points. Therefore, the algorithm processes spatial data more efficiently. Like DBSCAN, however, the algorithm cannot recognize clusters with varying densities. Uncu et al. [28] propose a three-step clustering algorithm (called GRIDBSCAN) to address the issue. The first step ensures homogeneous density in each grid by selecting appropriate grids. In the second step, cells that have similar densities are merged. Lastly, the DBSCAN algorithm is executed. However, these algorithms are not able to handle boundary regions between some adjacent clusters well. The main difference between the two algorithms and ours is the way they divide core and boundary nodes (or cells). The two algorithms apply a fixed, global density threshold to classify core and boundary nodes (or cells). In contrast, our algorithm uses an iterative boundary detection strategy to divide core and boundary nodes.

### 2.2. Density-Based Clustering

This subsection discusses several density-based clustering algorithms that are most relevant to our algorithm.

DBSCAN [19] is one of the most important density-based techniques. In DBSCAN, clusters are assumed to be connected to high-density regions separated by low-density regions. In DBSCAN, the core points are points in the dense part of a cluster. The boundary points in DBSCAN are defined as points that are part of a cluster but are not surrounded by a dense neighborhood. The primary difference between DBSCAN and our algorithm is that DBSCAN is a density-based clustering while our algorithm is a grid-based clustering. Our algorithm is similar to DBSCAN as it utilizes the notions of reachability and connectivity to find the maximally connected components of nodes. Despite this, they differ in their definitions of connectivity. DBSCAN defines connectivity between points and then generates clustering for points (including core points and boundary points) according to connectivity. However, our algorithm defines the connectivity between core nodes and then generates cluster cores for the core nodes based on their connectivity.

Rodriguez and Laio [20] propose density peaks clustering (DPC), a density-based algorithm. The algorithm assumes that cluster centers are surrounded by neighbors with lower local densities and that they are at a relatively large distance from any points with a higher local density. Our algorithm and DPC are similar in how the nodes (or points) are assigned. However, they are quite different. Unlike DPC’s assignment mechanism, where each non-centered point is assigned to the same cluster as its nearest neighbor with higher density, our assignment mechanism is a multilevel-based approach, where each boundary node is assigned to the same cluster as the node with the highest density among its nearest neighbors in the inner layers.

## 3. Related Concepts

### 3.1. DBSCAN Algorithm

DBSCAN [19] is one of the most widely used density-based clustering algorithms. It can identify arbitrary-shaped clusters and clusters with noise (i.e., outliers). In DBCSAN, there are two key parameters:

- ***ϵ* (or *eps*)**: It is a distance threshold. Two points are considered to be neighbors if the distance between them is less than or equal to *eps*.
- ***k* (or *minPts*)**: It specifies the minimum number of neighbors within a given radius.

Based on these two parameters, DBSCAN makes several definitions:

- **Core point**: A point is a core point if there are at least minPts number of points (including the point itself) in its surrounding area with radius eps.
- **Reachable**: A point $x_{q}$ is directly reachable from $x_{p}$ if point $x_{q}$ is within distance eps from core point $x_{p}$.
- **Density-connected**: Two points $x_{p}$ and $x_{q}$ are density-connected if $x_{p}$ is directly or transitively reachable from $x_{q}$ or $x_{q}$ is directly or transitively reachable from $x_{p}$.
- **Boundary point**: A point is a boundary point if it is reachable from a core point and there are less than minPts number of points within its surrounding area.
- **Noise point (or outlier)**: A point is a noise point (or outlier) if it is not a core point and not reachable from any core points.
- **Cluster**: A cluster w.r.t. eps and minPts is a non-empty maximal subset of the data set such that every pair of points in the cluster is density-connected.

**Figure 1:**
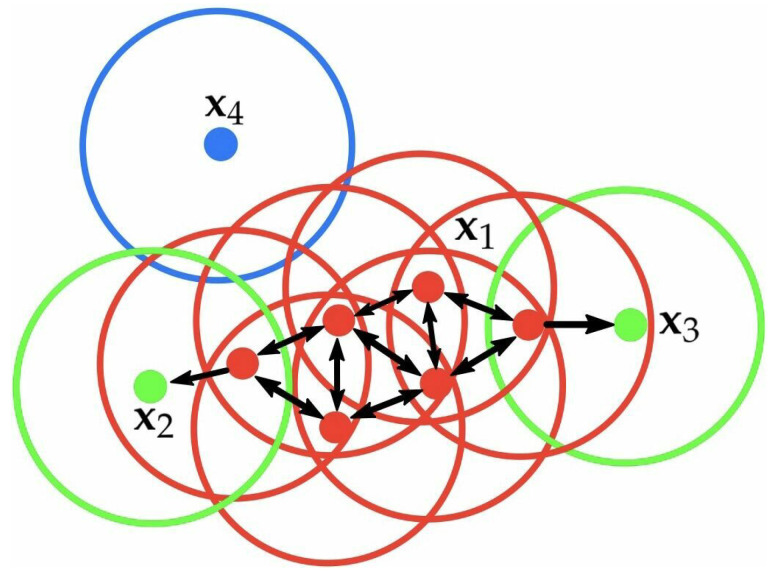
Example of some notions in DBSCAN.

To explain the notions of core point, boundary point, and noise point, we provide an example in Figure 1. Red points are core points because there are at least 4 points within their surrounding area with a radius of eps. This area is shown with the circles in the figure. The green points are boundary points because they are reachable from a core point and have less than 4 points within their neighborhood. Reachable means being in the surrounding area of a core point. The points $x_{2}$ and $x_{3}$ have two points (including the point itself) within their neighborhood (i.e., the surrounding area with a radius of eps). Finally, $x_{4}$ is a noise point because it is not a core point and cannot be reached from a core point.

### 3.2. DPC Algorithm

Rodriguez and Laio [20] present density peaks clustering (DPC), an algorithm that combines density-based clustering algorithms with centroid-based clustering algorithms. This algorithm has its basis on the assumptions that cluster centers are surrounded by neighbors with lower local densities and that they are far away from points of higher densities. There are two important quantities to describe each point $x_{i}$: its local density $\rho_{i}$ and its distance from the nearest larger density point $\delta_{i}$. The local density $\rho_{i}$ of $x_{i}$ is calculated as

(1) $$\rho_{i}=\sum_{x_{j}\in X}\chi \left(d\left(x_{i},x_{j}\right)-d_{c}\right),\chi \left(z\right)=\left\{\begin{array}{cc} 1, & z<0\\ 0, & z\geq 0 \end{array}\right.$$

where $d(x_{i},x_{j})$ is the distance between points $x_{i}$ and $x_{j}$, and the “cutoff distance” $d_{c}$ is a user-specified parameter.

For point $x_{i}$ with the highest density, DPC defines $\delta_{i}=max\left(d\left(x_{i},x_{j}\right)\right)$. For the other points, $\delta_{i}$ is the minimum distance between point $x_{i}$ and another point $x_{j}$ whose $\rho_{j}$ is higher than $\rho_{i}$. Its formula is as follows:

(2) $$\begin{array}{c} \delta_{i}=\min_{x_{j}:\rho_{j}>\rho_{i}}\left(d\left(x_{i},x_{j}\right)\right) \end{array}$$

where $x_{j}\in X$. X denotes the whole data set.

Based on DPC’s center assumption, density peaks with large ρ-δ are manually selected as centers by observing through a decision graph (i.e., a ρ-δ plot). Subsequently, each non-center point is allocated to the same cluster as its nearest point with higher density.

## 4. Proposed Algorithm

In this section, we describe our proposed clustering algorithm in detail and analyze its computational complexity.

### 4.1. Standard Grid Structure

As with most grid-based clustering algorithms, the first step in GCBD is to create a grid structure that divides the data space into a finite number of cells. To simplify subsequent calculations, we create a standard grid structure.

Let $X=\{x_{1},x_{2},\ldots ,x_{n}\}$ is a dataset consisting of *n* data points, where each data point has *m* features, i.e., $x_{i}=\left\{x_{i1},x_{i2},\ldots ,x_{im}\right\}$. ${\bar{x}}_{j}$ and ${\underline{x}}_{j}$ denote the maximum and minimum values of the *j*-th feature, respectively. Assume that each dimension should be divided into *l* equal intervals. Features in the original dataset are scaled to fall between 1 and l+1 by using the scaling function Φ(·).

(3) $$\Phi \left(x_{ij}\right)=\left(l\cdot \frac{x_{ij}-{\underline{x}}_{j}}{{\bar{x}}_{j}-{\underline{x}}_{j}}\right)+1$$

Let $A=A_{1}\times A_{2}\times \cdots \times A_{m}$ is the transformed feature space, where $A_{1},A_{2},\cdots ,A_{m}$ are the domains of the features (dimensions) of A. We define the notion of a standard grid structure.

**Definition** **1** (Standard grid structure). *A grid structure is called a standard grid structure, if the transformed feature space is divided into l intervals of equal length after each transformed feature is scaled by Equation (3).*

Let $v=\{v_{1},v_{2},\cdots ,v_{m}\}$ is a node in the standard grid structure. We will obtain the following property.

**Property** **1.** *The nodes in the standard grid structure are only located with integer coordinates, i.e., for each $v_{j}$, 1≤j≤m, $v_{j}\in \left\{1,2,\cdots ,l\;+\;1\right\}$.*

**Figure 2:**
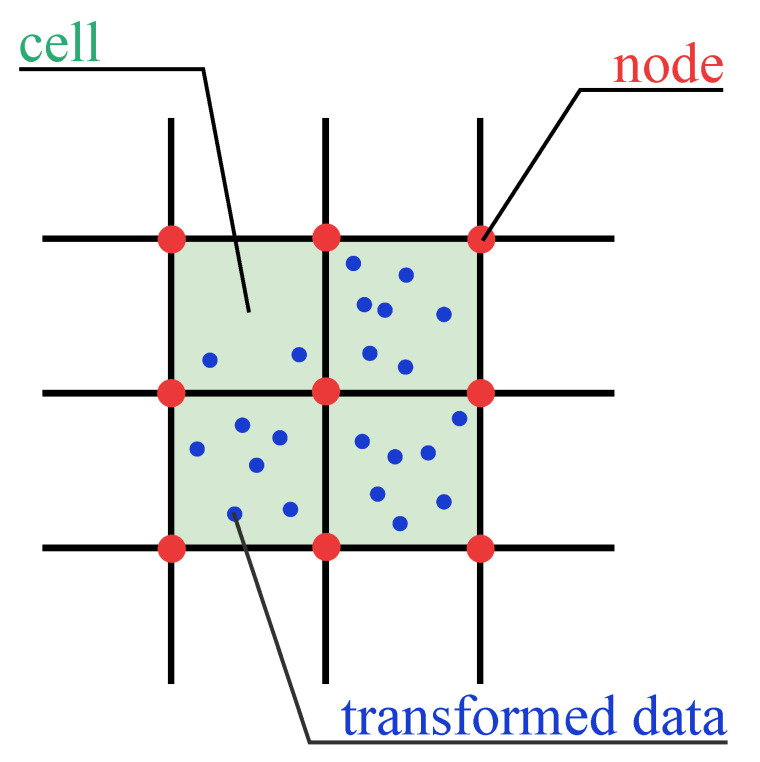
Example of some notions in GCBD.

To explain some notions in the standard grid structure, Figure 2 provides an example in a two-dimensional space. Green-shaded rectangles are cells in the standard grid structure. Red intersection points in the standard grid are called nodes. Blue points are transformed data.

### 4.2. Density Estimation

In density-based clustering, the densities of data points are computed. The traditional grid-based clustering calculates the densities of cells. In the proposed algorithm, we focus on the nodes’ density.

At the *t*-th iteration, we define $V^{\left(t\right)}$ as the set of active nodes and $X^{\left(t\right)}$ as the set of active points. To estimate the densities of nodes, we calculate the similarity between nodes and data points. In *j*-th dimension, a local scaling function of the node $v\in V^{\left(t\right)}$ and the data point $x_{i}\in X^{\left(t\right)}$ is given by

(4) $$f_{j}^{\left(t\right)}\left(v_{j}\right)=\max\left(1-\left|\Phi (x_{ij})-v_{j}\right|,0\right)$$

where $v_{j}$ is the coordinate of the grid node $v\in V^{\left(t\right)}$ in the *j*-th dimension and $x_{ij}$ is the coordinate of the data point $x_{i}\in X^{\left(t\right)}$ in the *j*-th dimension.

Using *f*, the node’s density value at *t*-th iteration is given by

(5) $$\begin{array}{c} \rho_{v}^{\left(t\right)}=\prod_{j=1}^{m}f_{j}^{\left(t\right)}\left(v_{j}\right) \end{array}$$

It is worth noting that each data point only affects the densities of the vertices (nodes) of the cell in which it is located. There may be a large number of nodes with a density value of 0 in the standard grid. To reduce the computational efficiency, we use a sparse tensor to preserve the density of non-zero nodes.

### 4.3. Boundary Detection

Inspired by border-peeling clustering [29], we use an iterative boundary detection strategy to divide the core and boundary nodes. The GCBD algorithm will classify a portion of the nodes of $V^{\left(t\right)}$ as boundary nodes and assign an inactive status to them during every boundary detection iteration.

The inactive nodes of $V^{\left(t\right)}$ are defined using a specific cut-off value, as follows:

(6) $$\begin{array}{c} V_{U}^{\left(t\right)}=\left\{v|\rho_{v}^{\left(t\right)}\leq \tau^{\left(t\right)}\right\} \end{array}$$

As with [16], a percentile is used to indirectly provide a series of cut-off values. The inactive nodes are defined as nodes whose density values fall below the 10-th percentile.

The inactive data points of $X^{\left(t\right)}$ are given by

(7) $$\begin{array}{c} X_{U}^{\left(t\right)}=\left\{x|dist_{C}\left(\Phi \left(x\right),v\right)\leq 1,v\in V_{U}^{\left(t\right)}\right\} \end{array}$$

where $dist_{C}$ is the Chebyshev distance.

At the next iteration, the active nodes are given by

(8) $$V^{\left(t\;+\;1\right)}=V^{\left(t\right)}\backslash V_{U}^{\left(t\right)}.$$

Similarly, the active nodes at the next iteration are given by

(9) $$X^{\left(t\;+\;1\right)}=X^{\left(t\right)}\backslash X_{U}^{\left(t\right)}.$$

At the end of all iterations, the set of activated nodes and the set of active data points are $V^{\left(T\;+\;1\right)}$ and $X^{\left(T\;+\;1\right)}$, where *T* is the number of iterations.

**Definition** **2** (Core node). *A node v is called a core node if it belongs to the set $V^{\left(T\;+\;1\right)}$, i.e., if $v\in V^{\left(T\;+\;1\right)}$.*

**Definition** **3** (Core point). *A point x is called a core point if it belongs to the set $X^{\left(T\;+\;1\right)}$, i.e., if $x\in X^{\left(T\;+\;1\right)}$.*

**Definition** **4** (Boundary node). *A node v is called a boundary node if it belongs to the set $V_{B}^{\left(T\right)}$, where $V_{B}^{\left(T\right)}=V_{U}^{\left(1\right)}\cup V_{U}^{\left(2\right)}\cup \cdots \cup V_{U}^{\left(T\right)}$, i.e., if $v\in V_{U}^{\left(1\right)}\cup V_{U}^{\left(2\right)}\cup \cdots \cup V_{U}^{\left(T\right)}$.*

**Definition** **5** (Boundary point). *A point x is called a boundary point if it belongs to the set $X_{B}^{\left(T\right)}$, where $X_{B}^{\left(T\right)}=X_{U}^{\left(1\right)}\cup X_{U}^{\left(2\right)}\cup \cdots \cup X_{U}^{\left(T\right)}$, i.e., if $x\in V_{U}^{\left(1\right)}\cup V_{U}^{\left(2\right)}\cup \cdots \cup V_{U}^{\left(T\right)}$.*

### 4.4. Connection Strategy

Next, we introduce the merging and assignment steps for nodes.

#### 4.4.1. Merging Step

Inspired by DBSCAN [19], we devise a merging step to classify the core nodes. We define the following notions.

**Definition** **6** (Reachable). *Two core nodes $v_{p}$ and $v_{q}$ are reachable if $dist_{C}(v_{p}$, $v_{q})\;=1$.*

**Definition** **7** (Connected). *Two core nodes $v_{p}$ and $v_{q}$ are connected if they are directly or transitively reachable.*

**Definition** **8** (Cluster core). *A Cluster core $\hat{C}$ is a non-empty maximal subset of $V^{\left(T\;+\;1\right)}$ such that every pair of nodes in $\hat{C}$ is connected.*

#### 4.4.2. Assignment Step

Inspired by DPC [20], we devise an assignment step to assign boundary nodes to clusters. For each node $v_{p}\in V_{U}^{\left(t\right)}$, we find a node $v_{q}\in V^{\left(t\;+\;1\right)}$ with the highest density among its nearest neighbors. We associate $v_{p}$ to $v_{q}$ and form the resulting clusters $C=\{C_{1},C_{2},\cdots ,C_{k}\}$.

#### 4.4.3. Mapping of Points to Clusters

All points are clustered into the same group with their nearest nodes. To improve computational efficiency, we use the “round” function to find the matching node for the point, instead of using the distance function to calculate distances between the point and all nodes.

### 4.5. Algorithm Description and Complexity Analysis

The proposed algorithm is summarized in Algorithm 1.
**Algorithm 1:** GCBD algorithm**Input**:    A set of points, $X=\{x_{1},\cdots ,x_{n}\}$                The number of intervals, *l*                The number of iterations, *T***Output**: The clustering result, $C=\{C_{1},C_{2},\cdots ,C_{k}\}$1 Create a standard grid structure based on Definition 1.2 Calculate the initial node density for each node according to Equation (5).3 Categorize nodes as core nodes or boundary nodes based on an iterative boundary detection strategy.4 Merge core nodes based on Definition 8.5 Assign boundary nodes (see Section 4.4.2).6 Map points to clusters (see Section 4.4.3).

We analyze the time complexity of GCBD. Assume that the number of points, the number of dimensions, and the number of intervals in each dimension are denoted by *n*, *m*, and *l*, respectively. The number of all nodes is *g*, and the number of sparse nodes is $g^{\prime }$. The number of iterations is *T*. A standard grid structure can be constructed in O(n) time. The time complexity of calculating the initial node density is O(gn). In the worst case, the time complexity of the third step is $O\left(T\left(g^{\prime 2}+g^{\prime }n\right)\right)=O\left(g^{\prime 2}+g^{\prime }n\right)$, where *T* is a small constant representing the number of iterations. The merging step and assignment step can be completed in $O(g^{\prime }\log\left(g^{\prime }\right))$ time. The time complexity of the last step is O(n).The time complexity of GCBD is approximated to be $O(g^{\prime }n)$ if $g^{\prime }\ll n$, $O(g^{\prime 2})$ otherwise.

We also give the time complexity of some existing algorithms. The time complexity of DGB [27] is $O(g^{2})$, where *g* is the number of nodes. The time complexity of WaveCluster [24] is O(c)≈O(g), where *c* is the number of cells. The time complexity of CLIQUE [25] is the number of $O\left(c^{2}\right)\approx O\left(g^{2}\right)$. The time complexity of BANG [22] is O(nlog(n)), where *n* is the number of points. The time complexity of GRIDBSCAN [28] is O(clog(c)). The time complexity of OpiGrid [26] is O(nlog(n)).

## 5. Experiments

In the following, the performance of the proposed clustering is studied in comparison to several grid-based clustering algorithms on various datasets.

### 5.1. Experiment Setup

We compare the performance of GCBD with 6 grid-based clustering algorithms, including DGB [27], WaveCluster [24], CLIQUE [25], BANG [22], GRIDBSCAN [28] and OpiGrid [26]. According to the following parameter settings, we search for the best clustering result. GCBD, DGB, WaveCluster, CLIQUE, and OpiGrid all require a parameter *l* describing the number of intervals. Its range is between [5,50]. In GCBD, the parameter *T* indicates the number of iterations. This parameter value lies in the range [2,12]. DGB and WaveCluster have one parameter ε. We set ε ranging from 0.01 to 0.1 with step 0.01. In WaveCluster and BANG, the parameter *h* indicates the number of levels. Its value falls between [1,5]. CLIQUE, BANG, and GRIDBSCAN all require a parameter *c* which indicates the density threshold. Its range is between [0,5]. GRIDBSCAN has one parameter *p*. We set *p* ranging from 0.01 to 0.1 with step 0.01.

We evaluate performance using 12 synthetic datasets, and six real-world datasets. The synthetic datasets include Mickey, Gu, Jain, ThreeD, DiffD, Moons, Shape3, Handl, Yinyang, T4, T7, and SF. The real-world datasets include ORL, Dermatology, Control, Dig, Optdigits, and Satimage. These datasets come from benchmark clustering datasets (https://github.com/milaan9/Clustering-Datasets, accessed on 1 November 2022) UCI machine learning repository (http://archive.ics.uci.edu/ml/index.php, accessed on 1 November 2022). Table 1 provides detailed information about these datasets.

Due to available class labels for the selected data sets, we employ external evaluation measures including Adjusted Mutual Information (AMI) [30], Fowlkes-Mallows index (FMI) [31], and F1 score [32]. The three measures range from [0,1], with 1 denoting perfect results and 0 denoting the worst ones.

### 5.2. Results on Synthetic Datasets

In Figure 3, Figure 4, Figure 5, Figure 6, Figure 7, Figure 8, Figure 9, Figure 10, Figure 11, Figure 12, Figure 13 and Figure 14, we present Mickey, Gu, Jain, ThreeD, DiffD, Moons, Shape3, Handl, Yinyang, T4, T7, and SF as examples to illustrate the superiority of our algorithm. Each cluster is indicated by a different color solid dot. And black dots with hollow shapes indicate noisy data.

**Figure 3:**
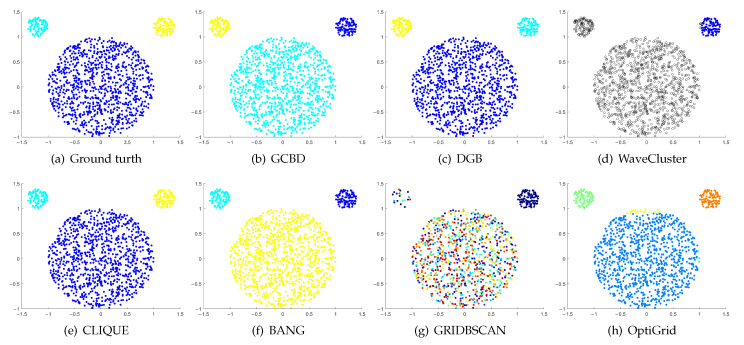
Clustering results on Mickey. (**a**) True structure of the Mickey dataset: It consists of three spherical clusters with different sizes. (**b**) Result of GCBD: It can yield the optimal structure of clusters. (**c**) Result of DGB: It can yield the optimal structure of clusters. (**d**) Result of WaveCluster: Two clusters are wrongly marked as outliers. (**e**) Result of CLIQUE: It can yield the optimal structure of clusters. (**f**) Result of BANG: It can yield the optimal structure of clusters. (**g**) Result of GRIDBSCAN: It yields over-partitioning. (**h**) Result of OptiGrid: A few points are erroneously clustered.

**Figure 4:**
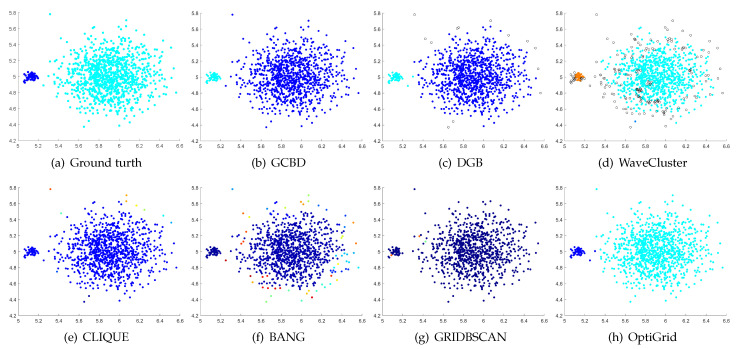
Clustering results on Gu. (**a**) True structure of the Gu dataset: It consists of two Gaussian clusters with different sizes. (**b**) Result of GCBD: It can yield the optimal structure of clusters. (**c**) Result of DGB: Very few points are erroneously clustered. (**d**) Result of WaveCluster: A few points are wrongly marked as outliers. (**e**) Result of CLIQUE: Two clusters are mistakenly merged into one. (**f**) Result of BANG: Two clusters are mistakenly merged into one. (**g**) Result of GRIDBSCAN: Two clusters are mistakenly merged into one. (**h**) Result of OptiGrid: Very few points are erroneously clustered.

In Figure 3 and Figure 4, the first two rows correspond to the clustering results of Mickey and Gu, two unbalanced data sets. The Mickey dataset is perfectly clustered by DGB, CLIQUE, BANG, and our algorithm. Compared to the Mickey dataset, the two spherical clusters on the Gu dataset are closer together, which makes clustering more challenging. Our algorithm is the only one that can cluster this dataset perfectly.

**Figure 5:**
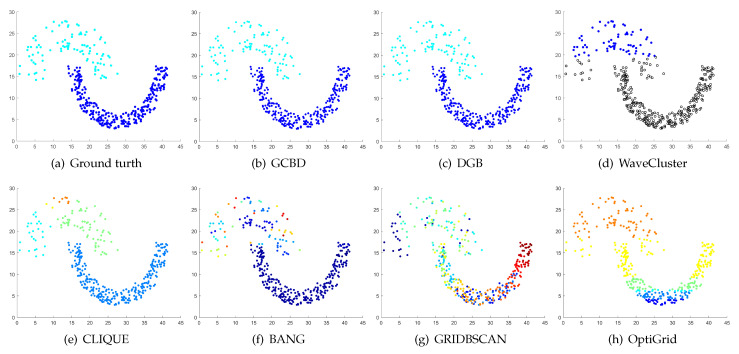
Clustering results on Jain. (**a**) True structure of the Jain dataset: It consists of two crescent-shaped clusters with different densities. (**b**) Result of GCBD: It can yield the optimal structure of clusters. (**c**) Result of DGB: It can yield the optimal structure of clusters. (**d**) Result of WaveCluster: Points in the cluster below are wrongly marked as outliers. (**e**) Result of CLIQUE: It divides the upper cluster into several small sub-clusters. (**f**) Result of BANG: It yields over-partitioning. (**g**) Result of GRIDBSCAN: It yields over-partitioning. (**h**) Result of OptiGrid: Two clusters are divided incorrectly into several small sub-clusters.

**Figure 6:**
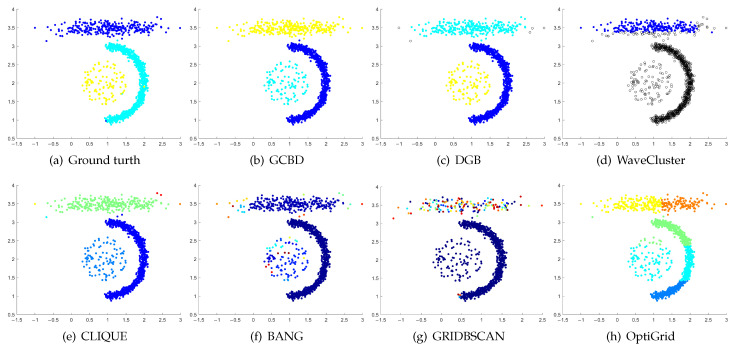
Clustering results on ThreeD. (**a**) True structure of the ThreeD dataset: It consists of three clusters with different densities. (**b**) Result of GCBD: It can correctly find the general shapes of each cluster. (**c**) Result of DGB: A few points are wrongly marked as outliers. (**d**) Result of WaveCluster: Two clusters are wrongly marked as outliers. (**e**) Result of CLIQUE: Very few points are erroneously clustered. (**f**) Result of BANG: A mass of points are erroneously clustered. (**g**) Result of GRIDBSCAN: It yields over-partitioning. (**h**) Result of OptiGrid: All clusters are divided incorrectly into several small sub-clusters.

**Figure 7:**
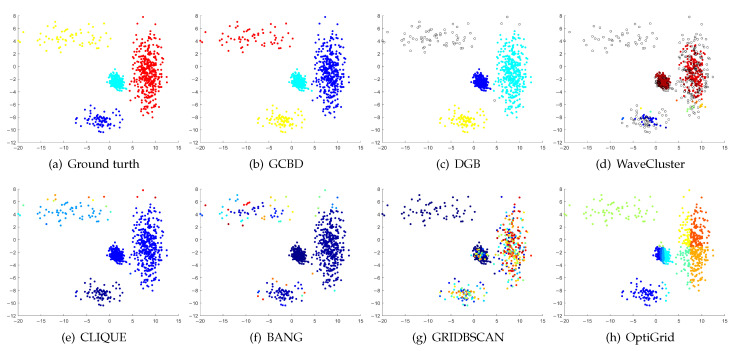
Clustering results on DiffD. (**a**) True structure of the DiffD dataset: It consists of four Gaussian clusters with different sizes. (**b**) Result of GCBD: It can correctly find the general shapes of each cluster. (**c**) Result of DGB: Points in the cluster upper are wrongly marked as outliers. (**d**) Result of WaveCluster: A mass of points are wrongly marked as outliers. (**e**) Result of CLIQUE: Two clusters are mistakenly merged into one. (**f**) Result of BANG: Two clusters are mistakenly merged into one. (**g**) Result of GRIDBSCAN: It yields over-partitioning. (**h**) Result of OptiGrid: Two clusters are divided incorrectly several small sub-clusters.

Figure 5, Figure 6 and Figure 7 show clustering results for three datasets with different densities (Jain, ThreeD, and DiffD). A perfect clustering of the Jain dataset is achieved by DGB and our algorithm. On the ThreeD dataset, the results of GCBD and DGB are nearly perfectly right. On the DiffD dataset, only our algorithm can find the correct number of clusters. Some algorithms (DGB and WaveCluster) incorrectly classify low-density clusters as noise, and others (CLIQUE and Bang) merge two adjacent high-density clusters into one class.

**Figure 8:**
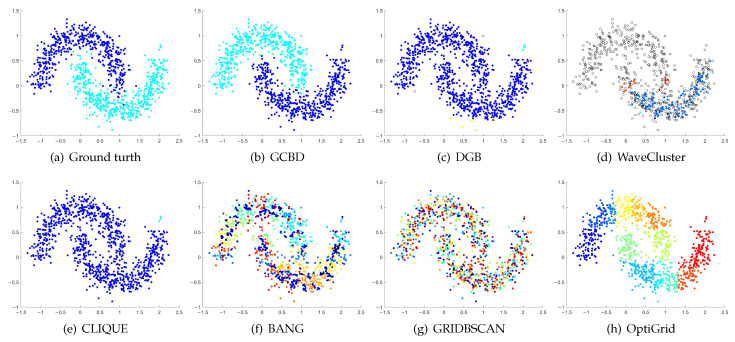
Clustering results on Moons. (**a**) True structure of the Moons dataset: It consists of two crescent-shaped clusters with different densities. (**b**) Result of GCBD: It can correctly find the general shapes of each cluster. (**c**) Result of DGB: Two clusters are mistakenly merged into one. (**d**) Result of WaveCluster: Points in the cluster upper are wrongly marked as outliers. (**e**) Result of CLIQUE: Two clusters are mistakenly merged into one. (**f**) Result of BANG: It yields over-partitioning. (**g**) Result of GRIDBSCAN: It yields over-partitioning. (**h**) Result of OptiGrid: Two clusters are divided incorrectly into several small sub-clusters.

**Figure 9:**
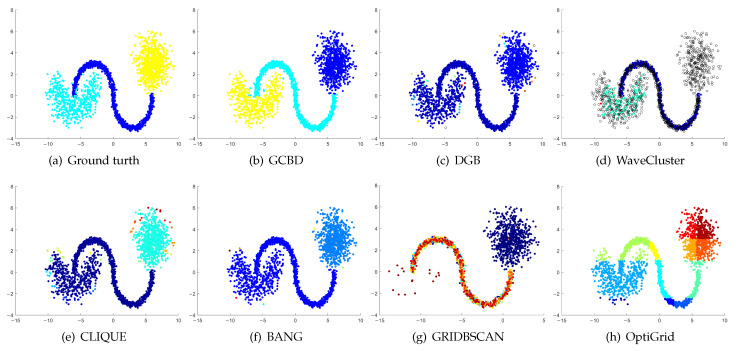
Clustering results on Shape3. (**a**) True structure of the Shape3 dataset: It consists of three clusters with different densities. (**b**) Result of GCBD: It can correctly find the general shapes of each cluster. (**c**) Result of DGB: Two clusters are mistakenly merged into one. (**d**) Result of WaveCluster: One cluster is wrongly marked as an outlier. (**e**) Result of CLIQUE: Two clusters are mistakenly merged into one. (**f**) Result of BANG: Two clusters are mistakenly merged into one. (**g**) Result of GRIDBSCAN: It yields over-partitioning. (**h**) Result of OptiGrid: Two clusters are divided incorrectly into several small sub-clusters.

**Figure 10:**
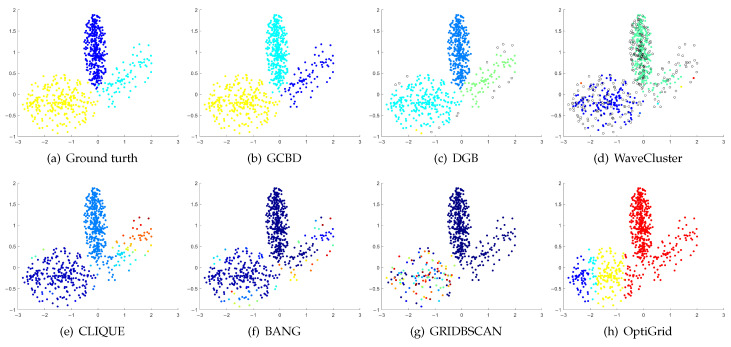
Clustering results on Handl. (**a**) True structure of the Handl dataset: It consists of three clusters with different densities. (**b**) Result of GCBD: It can correctly find the general shapes of each cluster. (**c**) Result of DGB: A few points are wrongly marked as outliers. (**d**) Result of WaveCluster: A mass of points are wrongly marked as outliers. (**e**) Result of CLIQUE: One cluster is divided incorrectly into several small sub-clusters. (**f**) Result of BANG: Three clusters are mistakenly merged into one. (**g**) Result of GRIDBSCAN: It yields over-partitioning. (**h**) Result of OptiGrid: Two clusters are mistakenly merged into one.

Figure 8, Figure 9 and Figure 10 show clustering results for three datasets that have clusters with overlapping regions (Moons, Shape3 and Handl). On these three datasets, only our algorithm can discover the overall structure of the clusters. Almost all comparison algorithms produce false merges between adjacent clusters.

**Figure 11:**
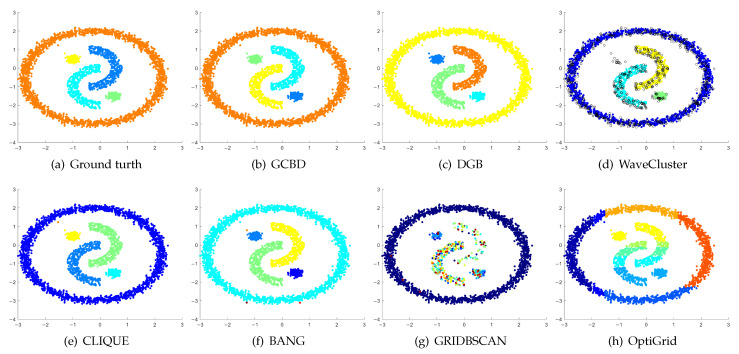
Clustering results on Yinyang. (**a**) True structure of the Yinyang dataset: Its composition is like a yin-yang diagram. (**b**) Result of GCBD: It can yield the optimal structure of clusters. (**c**) Result of DGB: It can yield the optimal structure of clusters. (**d**) Result of WaveCluster: A few points are wrongly marked as outliers. (**e**) Result of CLIQUE: Very few points are erroneously clustered. (**f**) Result of BANG: Very few points are erroneously clustered. (**g**) Result of GRIDBSCAN: It yields over-partitioning. (**h**) Result of OptiGrid: Three clusters are divided incorrectly into several small sub-clusters.

**Figure 12:**
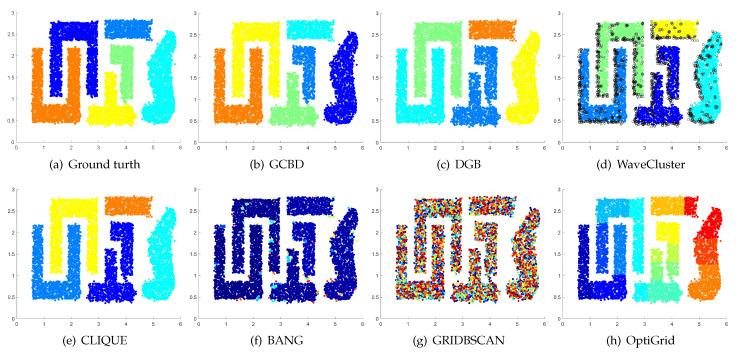
Clustering results on T4. (**a**) True structure of the T4 dataset: It consists of six intertwined or nested clusters. (**b**) Result of GCBD: It can yield the optimal structure of clusters. (**c**) Result of DGB: Two clusters are mistakenly merged into one. (**d**) Result of WaveCluster: A few points are wrongly marked as outliers. (**e**) Result of CLIQUE: Two clusters are mistakenly merged into one. (**f**) Result of BANG: Three clusters are mistakenly merged into one. (**g**) Result of GRIDBSCAN: It yields over-partitioning. (**h**) Result of OptiGrid: Six clusters are divided incorrectly several small sub-clusters.

**Figure 13:**
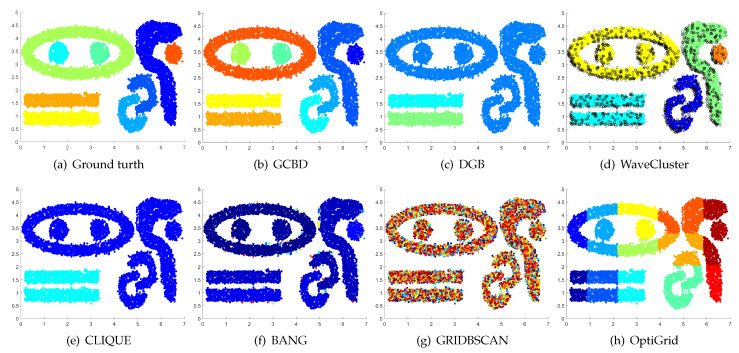
Clustering results on T7. (**a**) True structure of the T7 dataset: It consists of nine intertwined or nested clusters. (**b**) Result of GCBD: It can correctly find the general shapes of each cluster. (**c**) Result of DGB: Seven clusters are mistakenly merged into one. (**d**) Result of WaveCluster: Three clusters are mistakenly merged into one. (**e**) Result of CLIQUE: Seven clusters are mistakenly merged into one. (**f**) Result of BANG: Three clusters are mistakenly merged into one. (**g**) Result of GRIDBSCAN: It yields over-partitioning. (**h**) Result of OptiGrid: Five clusters are divided incorrectly several small sub-clusters.

**Figure 14:**
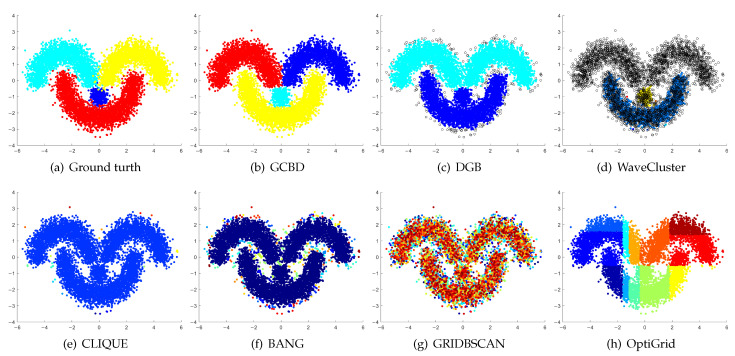
Clustering results on SF. (**a**) True structure of the SF dataset: These clusters look like smiling faces. (**b**) Result of GCBD: It can correctly find the general shapes of each cluster. (**c**) Result of DGB: Two clusters are mistakenly merged into one. (**d**) Result of WaveCluster: Two clusters are wrongly marked as outliers. (**e**) Result of CLIQUE: All clusters are mistakenly merged into one. (**f**) Result of BANG: All clusters are mistakenly merged into one. (**g**) Result of GRIDBSCAN: It yields over-partitioning. (**h**) Result of OptiGrid: Three clusters are divided incorrectly several small sub-clusters.

Figure 11, Figure 12, Figure 13 and Figure 14 correspond to the clustering results of four datasets with various shapes (Yinyang, T4, T7, and SF). The Yinyang dataset is perfectly clustered by DGB and our algorithm. On T4, T7, and SF, our algorithm outperforms the others.

Table 2 shows the quantitative results from these datasets. On all datasets, GCBD outperforms other algorithms in terms of all metrics. Experimental results show that our algorithm can identify clusters with different sizes, varying densities, overlapping regions, and arbitrary shapes.

### 5.3. Results on Real-World Datasets

We evaluate the clustering quality of all algorithms on 6 real-world datasets. The datasets are preprocessed the same way as in [33]. Table 3 illustrates that GCBD outperforms all comparison algorithms on most real-world datasets. For the high-dimensional dataset ORL, our algorithm improves F1 by an average of 0.30 compared to the others. Compared with DGB, WaveCluster, CLIQUE, BANG, GRIDBSCAN, and OptiGrid, GCBD has significantly better AMI and FMI. On the Dermatology dataset, our algorithm improves AMI and FMI by an average of 0.23 and 0.30 compared to the others. GCBD improves F1 by an average of 0.51 over WaveCluster, GRIDBSCAN, and OptiGrid. Furthermore, its F1 is far superior to DGB, CLIQUE, and BANG. On the Control dataset, GCBD has better AMI, FMI, and F1 than other algorithms. In particular, the improvement of F1 is more significant. On the Dig dataset, GCBD outperforms all comparison algorithms. On the Optdigits dataset, our algorithm has better AMI, FMI, and F1 than the others. Specifically, the F1 score of GCBD improves by an average of 0.36 over other algorithms. On the Satimage dataset, GCBD still achieves better clustering results. It improves the clustering AMI, FMI, and F1 by an average of 0.25, 0.30, and 0.32 compared to other algorithms. In summary, our algorithm improves AMI, FMI, and F1 by an average of 0.23, 0.27, and 0.29 over the others.

### 5.4. Running Time

This subsection compares the running times of GCBD and 6 competitors (DGB, WaveCluster, CLIQUE, BANG, GRIDBSCAN, and OptiGrid) on synthetic data sets with different sizes (*n* = 1000:1000:10,000). For a fair comparison, the number of intervals (a parameter common to GCBD, DGB, WaveCluster, CLIQUE, and OpiGrid) is kept at 20. To determine the running time, we use the average and standard deviation of 50 repeated experiments. We perform all experiments in the Matlab environment on a PC machine containing an Intel(R) Core(TM)-i7-9700F CPU and 32 GB RAM.

**Figure 15:**
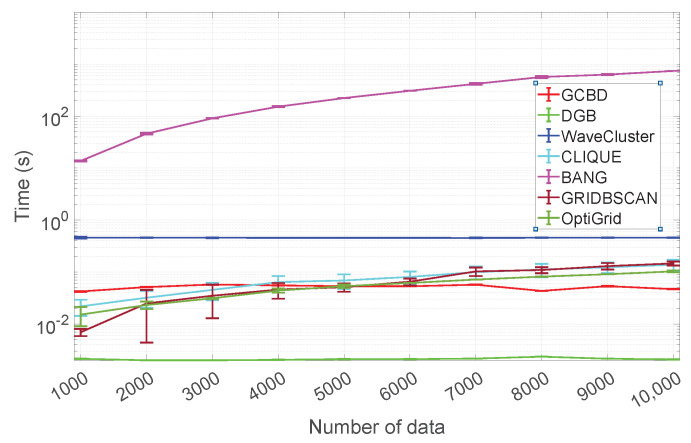
Running time comparison.

Figure 15 shows the average and standard deviation of the running times for GCBD, DGB, WaveCluster, CLIQUE, BANG, GRIDBSCAN, and OptiGrid. It is important to note that the y-axis is plotted using a base-10 log scale. We see that the BANG is significantly slower than other algorithms (GCBD, DGB, WaveCluster, CLIQUE, GRIDBSCAN, and OptiGrid). GCBD is the second-fastest in more than half of the cases. Scalability comparisons show our algorithm is competitive.

## 6. Conclusions

This paper presents a novel grid-based clustering algorithm for clusters with different sizes, varying densities, overlapping regions, and arbitrary shapes. Specifically, we define a density estimation of nodes based on a standard grid structure. We use an iterative boundary detection strategy to distinguish core nodes from boundary nodes. Therefore, the density threshold does not need to be specified by the user. In addition, the iterative density estimation and boundary detection can discover the boundary regions between adjacent clusters well, which facilitates the processing of clusters with varying densities and overlapping regions. Finally, the adopted connectivity strategy is beneficial for identifying clusters of arbitrary shapes.

This algorithm is mainly applied only to low-dimensional data. However, in the case of very high-dimensional data. To embed the data into a proper dimension, dimension reduction techniques such as Principal Component Analysis (PCA) and Uniform Manifold Approximation and Projection (UMAP) may be adopted. To be able to better cluster high-dimensional data, our future work is to introduce the idea of subspaces to alleviate the problem of the “curse of dimensionality”.

## Figures and Tables

**Table 1:** Datasets used in experiments.

Names	#Instances	#Features	#Classes
Mickey	1200	2	3
Gu	1050	2	2
Jain	373	2	2
ThreeD	1300	2	3
DiffD	863	2	4
Moons	1000	2	2
Shape3	2250	2	3
Handl	715	2	3
Yinyang	3200	2	5
T4	7326	2	6
T7	9208	2	9
SF	16,384	2	4
ORL	100	10,307	10
Dermatology	366	34	6
Control	600	60	6
Dig	1797	64	10
Optdigits	5620	64	10
Satimage	6435	36	6

**Table 2:** Performance comparison for 7 clustering algorithms on 12 synthetic datasets.

Algorithm	AMI	FMI	F1	Clu#	Parameter	AMI	FMI	F1	Clu#	Parameter
	Mickey(3 classes)					Gu(2 classes)				
GCBD	**1.000**	**1.000**	**1.000**	3	l = 13; T = 2	**1.000**	**1.000**	**1.000**	2	l = 29; T = 4
DGB	**1.000**	**1.000**	**1.000**	3	l = 8; ε = 0.1	0.816	0.985	0.985	2	l = 15; ε = 0.05
WaveCluster	0.666	0.911	0.907	1	l = 5; ε = 0.01; h = 4	0.399	0.850	0.840	4	l = 10; ε = 0.1; h = 1
CLIQUE	**1.000**	**1.000**	**1.000**	3	l = 30; c = 0	−0.010	0.943	0.942	10	l = 18; c = 4
BANG	**1.000**	**1.000**	**1.000**	3	h = 10; c = 4	0.501	0.922	0.919	49	h = 12; c = 5
GRIDBSCAN	0.014	0.090	0.019	1091	p = 0.01; c = 1	0.017	0.835	0.831	137	p = 0.02; c = 2
OptiGrid	0.932	0.981	0.981	5	l = 5	0.944	0.998	0.998	2	l = 50
	Jain(2 classes)					ThreeD(3 classes)				
GCBD	**1.000**	**1.000**	**1.000**	2	l = 26; T = 9	**0.980**	**0.996**	**0.996**	3	l = 42; T = 4
DGB	**1.000**	**1.000**	**1.000**	2	l = 18; ε = 0.1	0.961	0.994	0.994	3	l = 25; ε = 0.05
WaveCluster	0.586	0.887	0.884	1	l = 5; ε = 0.01; h = 4	0.595	0.852	0.845	1	l = 5; ε = 0.01; h = 4
CLIQUE	0.840	0.971	0.971	5	l = 18; c = 0	0.959	0.994	0.994	7	l = 25; c = 0
BANG	0.547	0.850	0.839	42	h = 12; c = 0	0.859	0.985	0.985	28	h = 12; c = 5
GRIDBSCAN	0.000		0.000	373	p = 0.08; c = 1	0.283	0.836	0.834	281	p = 0.03; c = 1
OptiGrid	0.410	0.507	0.453	6	l = 50	0.528	0.550	0.503	5	l = 4
	DiffD(4 classes)					Moons(2 classes)				
GCBD	**0.993**	**0.997**	**0.997**	4	l = 44; T = 8	**0.894**	**0.972**	**0.972**	2	l = 30; T = 8
DGB	0.981	0.993	0.993	3	l = 18; ε = 0.05	0.026	0.699	0.661	3	l = 18; ε = 0.1
WaveCluster	0.510	0.607	0.602	35	l = 15; ε = 0.01; h = 1	0.252	0.510	0.508	26	l = 40; ε = 0.05; h = 3
CLIQUE	0.636	0.716	0.682	19	l = 25; c = 1	0.012	0.705	0.665	2	l = 18; c = 3
BANG	0.862	0.971	0.971	43	h = 12; c = 0	0.205	0.077	0.012	512	h = 15; c = 4
GRIDBSCAN	0.131	0.407	0.364	539	p = 0.1; c = 1	0.000	0.055	0.012	924	p = 0.01; c = 1
OptiGrid	0.617	0.516	0.453	9	l = 3	0.464	0.402	0.285	13	l = 8
	Shape3(3 classes)					Handl(3 classes)				
GCBD	**0.957**	**0.984**	**0.984**	3	l = 40; T = 9	**0.960**	**0.988**	**0.988**	3	l = 29 = 31; T = 11
DGB	0.724	0.795	0.778	14	l = 50; ε = 0.05	0.914	0.978	0.978	4	l = 30; ε = 0.1
WaveCluster	0.447	0.519	0.519	16	l = 25; ε = 0.1; h = 2	0.392	0.571	0.568	10	l = 20; ε = 0.01; h = 2
CLIQUE	0.683	0.789	0.776	39	l = 50; c = 0	0.750	0.924	0.924	31	l = 30; c = 0
BANG	0.753	0.799	0.780	8	h = 12; c = 0	0.650	0.845	0.845	35	h = 12; c = 4
GRIDBSCAN	0.181	0.513	0.425	1538	p = 0.05; c = 1	0.166	0.655	0.655	240	p = 0.05; c = 1
OptiGrid	0.558	0.483	0.417	12	l = 4	0.491	0.705	0.700	4	l = 8
	Yinyang(5 classes)					T4(6 classes)				
GCBD	**1.000**	**1.000**	**1.000**	5	l = 41; T = 2	**1.000**	**1.000**	**1.000**	6	l = 44; T = 3
DGB	**1.000**	**1.000**	**1.000**	5	l = 30; ε = 0.05	0.955	0.943	0.941	5	l = 40; ε = 0.05
WaveCluster	0.672	0.701	0.688	6	l = 15; ε = 0.01; h = 1	0.695	0.662	0.662	5	l = 12; ε = 0.1; h = 1
CLIQUE	0.999	**1.000**	**1.000**	6	l = 30; c = 4	0.955	0.943	0.941	6	l = 50; c = 0
BANG	0.997	0.999	0.999	8	h = 12; c = 4	0.932	0.963	0.963	110	h = 15; c = 4
GRIDBSCAN	0.530	0.864	0.859	1311	p = 0.03; c = 1	0.002	0.100	0.081	5619	p = 0.01; c = 1
OptiGrid	0.568	0.464	0.387	10	l = 1	0.734	0.565	0.512	14	l = 1
	T7(9 classes)					SF(4 classes)				
GCBD	**0.997**	**0.999**	**0.999**	9	l = 49; T = 5	**0.955**	**0.986**	**0.986**	4	l = 38; T = 12
DGB	0.598	0.542	0.454	4	l = 50; ε = 0.1	0.064	0.524	0.458	11	l = 50; ε = 0.1
WaveCluster	0.623	0.612	0.610	5	l = 15; ε = 0.05; h = 1	0.431	0.475	0.475	39	l = 50; ε = 0.1; h = 3
CLIQUE	0.527	0.532	0.441	2	l = 50; c = 2	0.022	0.552	0.469	14	l = 50; c = 4
BANG	0.949	0.934	0.933	62	h = 15; c = 4	0.641	0.752	0.723	347	h = 15; c = 0
GRIDBSCAN	0.002	0.066	0.041	7839	p = 0.01; c = 1	0.000	0.022	0.003	3940	p = 0.01; c = 1
OptiGrid	0.611	0.405	0.380	12	l = 5	0.555	0.509	0.469	10	l = 5

**Table 3:** Performance comparison for 7 clustering algorithms on 6 real-world datasets.

Algorithm	AMI	FMI	F1	Clu#	Parameter	AMI	FMI	F1	Clu#	Parameter
	ORL(10 classes)					Dermatology(6 classes)				
GCBD	**0.955**	**0.947**	**0.946**	12	l = 46; T = 6	**0.930**	**0.946**	**0.946**	6	l = 38; T = 11
DGB	0.919	0.892	0.886	16	l = 40; ε = 0.05	0.926	0.896	0.891	5	l = 12; ε = 0.1
WaveCluster	0.493	0.410	0.292	1	l = 8; ε = 0.01; h = 4	0.745	0.747	0.739	3	l = 5; ε = 0.01; h = 3
CLIQUE	0.949	0.936	0.935	12	l = 20; c = 0	0.926	0.896	0.891	5	l = 18; c = 0
BANG	0.900	0.862	0.852	17	h = 12; c = 0	0.871	0.786	0.786	6	h = 12; c = 0
GRIDBSCAN	0.149	0.261	0.174	89	p = 0.02; c = 1	−0.001	0.000	0.000	364	p = 0.01; c = 1
OptiGrid	0.854	0.777	0.753	19	l = 2	0.747	0.609	0.550	15	l = 1
	Control(6 classes)					Dig(10 classes)				
GCBD	**0.860**	**0.773**	**0.748**	4	l = 9; T = 2	**0.917**	**0.905**	**0.905**	10	l = 41; T = 8
DGB	0.860	0.773	0.748	4	l = 8; ε = 0.01	0.899	0.863	0.863	11	l = 50; ε = 0.1
WaveCluster	0.621	0.575	0.497	1	l = 5; ε = 0.01; h = 3	0.664	0.459	0.448	10	l = 15; ε = 0.01; h = 1
CLIQUE	0.860	0.773	0.748	4	l = 40; c = 0	0.903	0.844	0.841	10	l = 40; c = 0
BANG	0.860	0.773	0.748	4	h = 12; c = 0	0.899	0.862	0.862	19	h = 15; c = 0
GRIDBSCAN	0.196	0.384	0.373	409	p = 0.05; c = 1	−0.001	0.015	0.005	1701	p = 0.01; c = 1
OptiGrid	0.592	0.435	0.416	17	l = 3	0.751	0.644	0.638	10	l = 2
	Optdigits(10 classes)					Satimage(6 classes)				
GCBD	**0.955**	**0.963**	**0.963**	10	l = 46; T = 8	**0.722**	**0.772**	**0.766**	5	l = 28; T = 9
DGB	0.922	0.871	0.868	10	l = 30; ε = 0.05	0.658	0.648	0.609	4	l = 30; ε = 0.1
WaveCluster	0.712	0.559	0.557	11	l = 20; ε = 0.01; h = 1	0.460	0.513	0.484	3	l = 10; ε = 0.1; h = 1
CLIQUE	0.859	0.733	0.710	8	l = 40; c = 2	0.613	0.600	0.540	3	l = 40; c = 5
BANG	0.894	0.819	0.813	12	h = 15; c = 0	0.613	0.600	0.540	3	h = 12; c = 0
GRIDBSCAN	0.000	0.021	0.008	5243	p = 0.01; c = 1	0.003	0.034	0.010	5999	p = 0.01; c = 1
OptiGrid	0.796	0.669	0.668	17	l = 5	0.507	0.470	0.470	11	l = 5

## Data Availability

All datasets used for this study have been deposited in the clustering benchmarks repository (https://github.com/milaan9/Clustering-Datasets, accessed on 1 November 2022) and UCI Machine learning Repository (http://archive.ics.uci.edu/ml/index.php, accessed on 1 November 2022).
